# Changes in and predictors of length of stay in hospital after surgery for breast cancer between 1997/98 and 2004/05 in two regions of England: a population-based study

**DOI:** 10.1186/1472-6963-9-202

**Published:** 2009-11-09

**Authors:** Amy Downing, Mark Lansdown, Robert M West, James D Thomas, Gill Lawrence, David Forman

**Affiliations:** 1 Centre for Epidemiology & Biostatistics, Room 8.49 Worsley Building, University of Leeds, Leeds, LS2 9LN, UK; 2 Department of Surgery, The General Infirmary at Leeds, Leeds, LS1 3EX, UK; 3 West Midlands Cancer Intelligence Unit, Public Health Building, University of Birmingham, Edgbaston, Birmingham, B15 2TT, UK; 4 Northern & Yorkshire Cancer Registry & Information Service, Level 6 Bexley Wing, St James' Institute of Oncology, St James' University Hospital, Leeds, LS9 7TF, UK

## Abstract

**Background:**

Decreases in length of stay (LOS) in hospital after breast cancer surgery can be partly attributed to the change to less radical surgery, but many other factors are operating at the patient, surgeon and hospital levels. This study aimed to describe the changes in and predictors of length of stay (LOS) in hospital after surgery for breast cancer between 1997/98 and 2004/05 in two regions of England.

**Methods:**

Cases of female invasive breast cancer diagnosed in two English cancer registry regions were linked to Hospital Episode Statistics data for the period 1^st ^April 1997 to 31^st ^March 2005. A subset of records where women underwent mastectomy or breast conserving surgery (BCS) was extracted (n = 44,877). Variations in LOS over the study period were investigated. A multilevel model with patients clustered within surgical teams and NHS Trusts was used to examine associations between LOS and a range of factors.

**Results:**

Over the study period the proportion of women having a mastectomy reduced from 58% to 52%. The proportion varied from 14% to 80% according to NHS Trust. LOS decreased by 21% from 1997/98 to 2004/05 (LOSratio = 0.79, 95%CI 0.77-0.80). BCS was associated with 33% shorter hospital stays compared to mastectomy (LOSratio = 0.67, 95%CI 0.66-0.68). Older age, advanced disease, presence of comorbidities, lymph node excision and reconstructive surgery were associated with increased LOS. Significant variation remained amongst Trusts and surgical teams.

**Conclusion:**

The number of days spent in hospital after breast cancer surgery has continued to decline for several decades. The change from mastectomy to BCS accounts for only 9% of the overall decrease in LOS. Other explanations include the adoption of new techniques and practices, such as sentinel lymph node biopsy and early discharge. This study has identified wide variation in practice with substantial cost implications for the NHS. Further work is required to explain this variation.

## Background

The average time spent in hospital after treatment has been decreasing for many years. Data show that in the UK the average length of stay (LOS) for patients receiving acute care decreased from 19.8 days in 1953 to 8.8 days in 1982[1]. More recent data show that the decrease has continued and by 2005 the average LOS was 6.1 days[2]. Similar reductions have been seen in most developed countries.

LOS can vary according to a wide range of factors, including patient, physician and hospital characteristics [3-5]. Patient characteristics associated with increased hospital stays include older age, being unmarried or having no immediate family, lower socioeconomic status, more severe illness and presence of comorbidity [4-6]. Physician characteristics, such as age, level of experience and number of cases treated per year may have an effect on LOS. For example, younger, less experienced doctors may keep patients in hospital for longer than their older, more experienced colleagues[7]. Alternatively, those doctors trained more recently may opt to use less invasive techniques leading to shorter hospital stays. Finally, hospital characteristics, including hospital size or number of beds available and hospital discharge policy are likely to have an effect on LOS. In addition, the hospital-level LOS will be influenced by the population living within it's catchment area; if the population is more deprived and has a higher level of pre-existing illness then the overall LOS is likely to be longer[3,6].

The reductions in LOS for breast cancer patients appear to have been even greater. A study carried out in Canada reported that the average LOS for breast cancer patients undergoing surgery in the 1980s was approximately 3 days longer than that for all surgery patients combined, but by 2000 the average LOS for breast cancer patients was half the overall average[8]. Data from 22 European countries show substantial decreases in the average LOS for breast cancer patients between 1990 and 2005; the UK average decreased from 9.8 days in 1990 to 5.2 days in 2005[9].

One of the major changes in the treatment of breast cancer in recent decades is the change from mastectomy to breast conserving surgery (BCS), which is a less radical operation and therefore generally requires less time in hospital afterwards. In a study of Swedish breast cancer patients, Lindqvist et al estimated that 14% of decline seen in LOS could be attributed to the change from mastectomy to BCS[10]. At the same time there has been a move towards less invasive axillary surgery (from axillary node dissection to sampling and, more recently, sentinel lymph node biopsy [SLNB]), which may also explain some of the reduction in LOS. A further recent development is that of early discharge, where women leave hospital one or two days after surgery, and in some cases on the same day, with wound drains still in place, after it was shown that such practice did not result in increased rates of physical or psychological illness[11]. Other factors affecting LOS may include changes in casemix (towards younger patients with less advanced disease), the increasing specialisation of surgeons, and hospital policy in response to financial pressures.

Many health data, such as those used to investigate trends in surgical treatment and LOS, have an inherent clustering. For example, patients are managed by surgeons, who in turn are clustered within hospitals. Failure to account for this clustering can lead to bias in the resulting estimates; there may be effects due to variation in a surgeon's willingness to discharge early or aspects of the hospital resources. By using multilevel modelling it is possible to obtain more accurate estimates and in addition the amount of variation attributable to each level can be estimated. This methodology was used by Westert et al when looking at variations in duration of hospital stay for four (non-cancer) operations[12]. They found that the variation between hospitals was much larger than that within hospitals (i.e. between doctors). However, the breast cancer focused studies mentioned above used only standard regression techniques.

The aim of this study was to describe the changes in and predictors of LOS in hospital after surgery for breast cancer between 1997/98 and 2004/05 in two regions of England using multilevel modelling.

## Methods

### The study dataset

Cases of female invasive breast cancer diagnosed in the Northern & Yorkshire and West Midlands cancer registry regions between 1997 and 2005 were identified from the respective cancer registry databases. These two registries have high quality staging and treatment data and cover approximately 23% of the population of England, 35 of the 168 Acute NHS Trusts in the country and are broadly representative in terms of population structure and level of deprivation. The cancer registry data were linked (using all or combinations of the identifiers of NHS number, date of birth and postcode at diagnosis) to an extract of Hospital Episode Statistics (HES) data covering the time period of April 1997 to March 2005. The study team have been granted MREC and NIGB approvals to hold and link the data.

A subset of the linked records where women underwent mastectomy or BCS was extracted (n = 44 877). Where women underwent both types of operation, the record relating to the most radical surgery (mastectomy) was chosen. Where women underwent the same operation on more than one occasion the first operation was chosen. Patient age and stage of disease were obtained from the cancer registry data. A measure of comorbidity was derived using non-breast cancer diagnoses recorded in the year before surgery (in any of the 14 diagnosis codes in HES) according to the Charlson Index[13]. Using the operation codes it was possible to identify women who underwent axillary lymph node sampling/dissection or reconstructive/other plastic surgery to the breast at the same time as the main mastectomy or BCS procedure. Undergoing more than one procedure may lead to a longer hospital stay.

Information regarding the managing NHS Trust and the consultant in charge of each patient's care was derived from the HES episode that detailed the chosen surgical procedure. Consultant workloads were calculated as the median number of cases treated per year. These were divided into three groups; >= 40 cases, 41-80 cases and 81 or more cases, based on the premise that surgeons work 40 weeks per year and so the groups relate to those operating on less than one, between one and two, and more than two patients per week. No other consultant-level information is available from cancer registry or HES data (such age, or years since qualification) and is not freely available from other routine data sources. Information on the number of beds in each hospital (as an indicator of size) was obtained from the Department of Health Hospital Activity Statistics (number of general & acute beds available, excluding neonates and children)[14]. These were divided in to three roughly equal groups; less than 400, 400-799, 800 or more.

LOS was calculated as the difference between the admission date and discharge date. This was done using spells rather than episodes, as a patient can have several finished consultant episodes (FCEs) within one spell. For example, they may have one FCE for the operation and initial monitoring (lasting 2 days) and another FCE whilst they recover in a different ward (lasting 3 days) giving a total LOS of 5 days. LOS ranged from 0 to 170 days. There were 186 cases where a patient underwent a mastectomy and had a hospital spell of less than two days; these were excluded, as these patients are likely to have been transferred elsewhere but this has not been recorded. In some hospitals, where a policy of early discharge has been adopted, women undergoing BCS may have stays of less than 2 days and so these records were included (3445 cases). Cases operated on by a consultant with a workload of less than 10 per year were excluded to improve the reliability of the LOS variable (these were mainly cases where the consultant was incorrectly recorded as someone other than the surgeon, such as the anaesthetist). This left 42 611 records for analysis. Variations in age at surgery, tumour stage, comorbidities, lymph node sampling/dissection, reconstructive/plastic surgery to the breast(s), consultant workload, number of hospital beds and median LOS after surgery (the data were positively skewed) by operation type, year and region were investigated.

### Statistical analysis

The data structure is clustered with patients nested within surgical teams within NHS Trusts. To account for this clustering we used multilevel poisson regression, using the software MLwiN[15], which allows covariates (explanatory variables) to be incorporated in the model 'operating' at the correct level of the system hierarchy. The model was developed patients (level 1) clustered within surgical teams (identified by the consultant surgeon) (level 2) and NHS Trusts (level 3). The use of a Poisson model allowed LOS to be transformed to a log scale, thereby accounting for the skewed nature of the data. The resulting estimates were exponentiated and presented as ratios, where values <1.00 indicate a decreased LOS, values >1.00 indicate an increased LOS and values of 1.00 indicate no change in LOS according to the variable of interest.

The variables included in the model were operation type (mastectomy/BCS), year of diagnosis, age (per 10 year increase), stage at diagnosis (I, II, III, IV, missing), comorbidity score (0, 1+), lymph node excision (yes/no), reconstructive/plastic surgery (yes/no) and region (all specified at the patient level), consultant workload (10-40, 41-80, 81+) specified at the surgical team level and number of hospital beds (<400, 400-799, 800+) specified at the Trust level. When the model has run the total outcome variation is partitioned into that between patients but within surgical teams and that between patients but within Trusts, which indicates to what extent the unexplained variation within the model pertains to differences across surgeons or Trusts.

## Results

**Figure 1:**
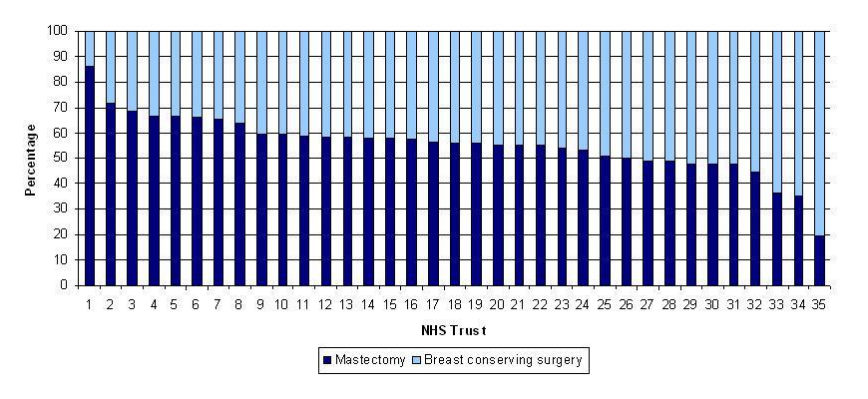
**Proportion of surgically treated women undergoing mastectomy and breast conserving surgery by NHS Trust (1997/98-2004/05)**. NB: The NHS Trusts are numbered from highest to lowest percentage of mastectomies; the numbers in Figure 1 do not necessarily correspond with those in Figures 2 and 3.

Table 1 shows the characteristics of the study population between 1997/98 and 2004/05 according to type of surgery (mastectomy or BCS). Overall 24 734 out of 44 877 (55.1%) of women undergoing surgery had a mastectomy and, over the study period, the proportion reduced from 58.4% to 52.3%. There was wide variation by NHS Trust in the proportion of mastectomies, varying from 13.7% to 80.2% (Figure 1). The average age at surgery was 60 years for the mastectomy patients and 58 years for the BCS patients and this remained relatively stable over the study period. The proportion with early stage (I/II) disease was 85.9% in the mastectomy group and 97.1% in the BCS group (excluding those with missing stage, which decreased over time) and this remained fairly constant over the period of study. Overall 66.3% of the mastectomy group and 78.8% of the BCS group had no comorbidities and this decreased from 75.6% to 60.8% and from 84.6% to 75.2% in the two groups over the 8 years. Conversely, those scoring 4 or more increased from 19.9% to 30.2% (26.8% overall) in the mastectomy patients and from 11.2% to 15.8% (14.8% overall) in the BCS patients. The majority of patients underwent some form of axillary node excision during their operation (88.7% of mastectomy patients and 87.5% of BCS patients) and this increased over time. The proportions undergoing reconstructive or other plastic surgery to the breast(s) during their operation were 15.1% and 2.8% of the mastectomy and BCS groups respectively, and this also increased slightly over the study period.

**Table 1:** Characteristics and length of stay of breast cancer patients between 1997/98 and 2004/05 by surgery group

**Variable**	**Group**	**All**	**1997/98**	**1998/99**	**1999/00**	**2000/01**	**2001/02**	**2002/03**	**2003/04**	**2004/05**
**No. patients**	**Mastectomy**	24,734	2,309	2,905	3,102	3,104	3,192	3,291	3,399	3,432
	**BCS**	20,143	1,646	2,170	2,333	2,391	2,588	2,803	3,077	3,135
	**All surgical**	44,877	3,955	5,075	5,435	5,495	5,780	6,094	6,476	6,567
										
**% patients**	**Mastectomy**	55.1	58.4	57.2	57.1	56.5	55.2	54.0	52.5	52.3
	**BCS**	44.9	41.6	42.8	42.9	43.5	44.8	46.0	47.5	47.7
										
**Average age (years)**	**Mastectomy**	60.7	60.4	60.0	60.4	60.4	61.1	60.8	61.6	60.9
	**BCS**	58.8	59.1	58.3	58.3	58.2	58.5	59.0	59.2	59.2
										
**Stage I (%)**	**Mastectomy**	25.3	19.3	24.6	25.7	26.0	26.0	25.8	25.7	27.6
	**BCS**	53.3	41.3	53.5	51.8	54.4	53.7	54.8	55.0	56.7
**Stage II (%)**	**Mastectomy**	53.7	39.1	55.7	54.7	55.6	55.8	54.1	55.3	55.3
	**BCS**	38.0	32.3	38.0	40.3	38.8	39.1	37.6	38.1	37.9
**Stage III (%)**	**Mastectomy**	11.3	9.2	11.2	11.6	10.2	11.0	12.4	12.5	11.7
	**BCS**	2.0	1.9	2.0	2.3	1.8	2.0	2.1	1.9	1.7
**Stage IV (%)**	**Mastectomy**	1.7	1.4	1.2	1.6	1.7	2.1	1.8	1.8	1.5
	**BCS**	0.7	0.4	0.9	0.9	0.9	0.7	0.8	0.8	0.3
**Stage missing (%)**	**Mastectomy**	8.0	31.1	7.2	6.4	6.5	5.1	5.9	4.7	3.9
	**BCS**	6.0	24.2	5.5	4.7	4.1	4.5	4.6	4.3	3.4
										
**Charlson 0 (%)**	**Mastectomy**	66.3	75.6	71.0	68.0	67.3	64.8	63.5	63.2	60.8
	**BCS**	78.8	84.6	81.6	80.8	80.2	79.5	77.2	75.7	75.2
**Charlson 1-3 (%)**	**Mastectomy**	6.8	4.5	5.7	6.7	6.7	6.4	6.9	7.9	9.0
	**BCS**	6.5	4.1	5.3	5.8	6.1	5.4	6.4	7.3	9.0
**Charlson 4+ (%)**	**Mastectomy**	26.8	19.9	23.3	25.3	26.0	28.7	29.5	28.9	30.2
	**BCS**	14.8	11.2	13.1	13.4	13.8	15.1	16.5	17.1	15.8
										
**Lymph node excision (%)**	**Mastectomy**	88.7	78.9	84.6	87.6	87.6	89.2	91.4	92.7	94.1
	**BCS**	87.5	77.4	82.5	84.0	85.2	89.1	90.1	91.7	92.7
										
**Reconstructive/plastic surgery (%)**	**Mastectomy**	15.1	11.8	13.4	15.3	14.9	15.8	16.9	15.7	15.8
	**BCS**	2.8	1.5	2.5	2.8	3.0	3.1	2.6	2.8	3.3
										
**Median length of stay (days)**	**Mastectomy**	5	6	6	6	5	5	5	5	5
	**BCS**	3	3	3	4	4	3	3	3	3

**Figure 2:**
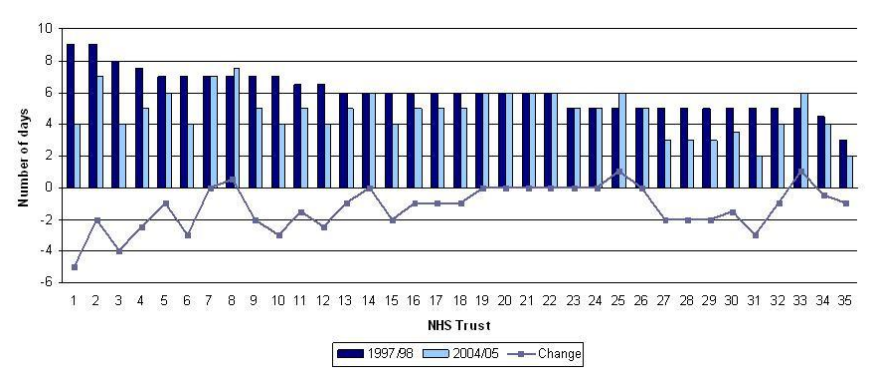
**Median LOS for patients undergoing mastectomy in 1997/98 and 2004/05 and the change over the study period by NHS Trust**. NB: The NHS Trusts are numbered from highest to lowest LOS; the numbers in Figure 2 do not necessarily correspond with those in Figures 1 and 3.

**Figure 3:**
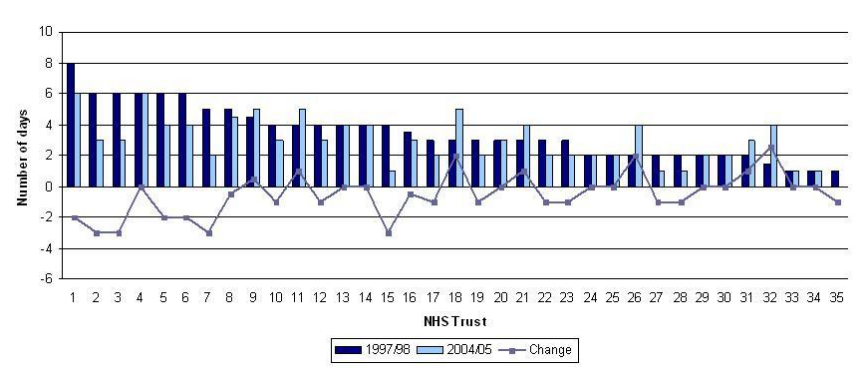
**Median LOS for patients undergoing breast conserving surgery in 1997/98 and 2004/05 and the change over the study period by NHS Trust**. NB: The NHS Trusts are numbered from highest to lowest LOS; the numbers in Figure 3 do not necessarily correspond with those in Figures 1 and 2.

Median LOS decreased from 5 to 4 days for all surgery patients; it decreased from 6 to 5 days for mastectomy patients and remained at 3 days for BCS patients (Table 1). The same pattern was observed in both regions. Figures 2 and 3 show the median LOS for each Trust at the start of the study period (1997/98) and at the end (2004/05) along with the overall change between the two periods for the mastectomy and BCS patients respectively. LOS after mastectomy decreased in 23 of the 35 Trusts, whilst LOS after BCS decreased in 18 Trusts.

In univariable analyses having a mastectomy, increasing age, advanced stage, presence of comorbidity, undergoing reconstructive/plastic surgery, undergoing lymph node excision and low consultant workload were associated with increased LOS. LOS did not differ according to region or number of hospital beds available and these variables were dropped from further analyses.

Table 2 shows the results of the multivariable analyses. When looking at LOS after the initial surgical procedure, undergoing BCS was associated with 33% shorter stays compared to undergoing mastectomy (LOS Ratio [LOSR] = 0.67, 95%CI 0.66-0.68). Hospital stays were 21% shorter in 2004/05 compared to 1997/98 (LOSR = 0.79, 95%CI 0.77-0.80). Older age was associated with an increased LOS (11% increase [LOSR = 1.11, 95%CI 1.11-1.11] per 10 year increase in age), as were advanced disease (18% increase [LOSR = 1.18, 95%CI 1.14-1.23] for patients with stage IV compared to stage I disease) and presence of comorbidities (8% increase [LOSR = 1.08, 95%CI 1.07-1.09] compared to no comorbidities). Undergoing lymph node excision and reconstructive/plastic surgery increased hospital stay by 8% each (LOSR = 1.08, 95%CI 1.07-1.10 and LOSR = 1.08, 95%CI 1.06-1.09 respectively). Consultant workload had no significant association with LOS (LOSR = 0.96, 95%CI 0.89-1.02 and LOSR = 0.99, 95%CI 0.87-1.13 for medium and high workloads compared to low workload).

**Table 2:** Associations between LOS and patient, treatment and organisational factors

	**Initial surgery**	**30 days post-op**
	**LOS Ratio (95% CI)**	**LOS Ratio (95% CI)**
		
**Mastectomy**	1.00	1.00
**BCS**	0.67 (0.66-0.68)	0.66 (0.66-0.67)
		
**1997/98**	1.00	1.00
**1998/99**	0.96 (0.94-0.98)	0.92 (0.91-0.94)
**1999/00**	0.95 (0.93-0.97)	0.92 (0.90-0.94)
**2000/01**	0.93 (0.91-0.95)	0.89 (0.88-0.91)
**2001/02**	0.90 (0.89-0.92)	0.87 (0.85-0.89)
**2002/03**	0.87 (0.85-0.89)	0.85 (0.84-0.87)
**2003/04**	0.85 (0.83-0.86)	0.84 (0.82-0.86)
**2004/05**	0.79 (0.77-0.80)	0.78 (0.76-0.80)
		
**Age (per 10 years)**	1.11 (1.11-1.11)	1.08 (1.08-1.08)
		
**Stage I**	1.00	1.00
**Stage II**	1.06 (1.05-1.07)	1.08 (1.07-1.09)
**Stage III**	1.14 (1.13-1.16)	1.15 (1.13-1.17)
**Stage IV**	1.18 (1.14-1.23)	1.20 (1.15-1.24)
**Missing**	1.04 (1.02-1.06)	1.03 (1.01-1.05)
		
**Comorbidities - No**	1.00	1.00
**Comorbidities - Yes**	1.08 (1.07-1.09)	1.08 (1.07-1.10)
		
**Nodes dissected - No**	1.00	1.00
**Nodes dissected - Yes**	1.08 (1.07-1.10)	1.13 (1.11-1.14)
		
**Reconstructive surg - No**	1.00	1.00
**Reconstructive surg - Yes**	1.08 (1.06-1.09)	1.08 (1.06-1.10)
		
**Consultant workload - 10-40**	1.00	1.00
**Consultant workload - 41-80**	0.96 (0.89-1.02)	0.96 (0.89-1.03)
**Consultant workload - 81+**	0.99 (0.87-1.13)	0.99 (0.90-1.09)
		
	**Variance (95% CI)**	**Variance (95% CI)**
		
**Trust level**	0.08 (0.04-0.13)	0.06 (0.03-0.10)
**Surgical team level**	0.05 (0.03-0.06)	0.04 (0.03-0.06)

The variance terms refer to the amount of residual (unexplained) variation at each of the levels. A figure of zero, or close to zero, means that there is little or no residual variation, i.e. all the variation at that level has been explained. The results show that a small amount of variation remained at the Trust and surgical team levels; 65.1% of this was attributed to the Trust level and 34.9% to the surgical team level. Comparison of this model to one with no covariates (null model; not shown) shows that 31.4% of the residual upper-level variation was explained by our model.

In addition to measuring LOS for the initial surgical procedure (from the date of admission to the date of discharge), we also calculated the total number of days spent in hospital for breast cancer related conditions from the date of operation to 30 days post operation (initial surgery plus any subsequent admissions for a subset of conditions including chemotherapy session, wound infection, haematoma, cellulitis, pulmonary embolism, problem related to implant/graft). The median LOS was 4 days. The proportion of women with a subsequent admission for one of the related conditions was 12.6%; the majority of these admissions were for chemotherapy (62.0%), followed by 'infection related to a procedure' (12.2%). The multilevel regression results were very similar to the results for the initial surgery alone (Table 2). The largest difference was seen for patients undergoing lymph node excision where the association strengthened to 1.13 (95%CI 1.11-1.14). The level-specific variances were also similar to the results for the initial surgery alone; 58.1% was attributed to the Trust level and 48.9% to the surgical team level. Compared to the null model (not shown) 34.8% of the residual upper-level variation was explained by the model with covariates.

## Discussion

In this study women undergoing BCS had 33% shorter hospital stays than women having a mastectomy. Over the 8 years the proportion of women undergoing mastectomy decreased from 58% to 52%. During this time there was a reduction in LOS of 21%, equivalent to a decrease of one day (the median LOS at the start of the study was 5 days). However, the change from mastectomy to BCS accounts for only 9% of the observed decrease in LOS. Another possible reason for the reduction in LOS is an increase in the number of women undergoing SLNB instead of axillary node dissection (which is less invasive, thus requiring less recovery time); however, we are unable to look in to this further, as SNLB is a relatively new technique and is not yet routinely coded in HES data. Some of these patients may have been subsequently re-admitted for completion of axillary node dissection after finding involved nodes in the SLNB, but these should have been picked up in our secondary analysis of the 30 day post-operation period. A further explanation is an increase in the number of women being admitted on the day of their operation rather than the day before. Over the study period this increased from 29% to 42% of patients, and thus may account for some of the reduction in LOS. Other reasons include increased adoption of early discharge and a change in the criteria used to assess when women are fit for discharge, however, the measurement of such policy factors would involve the collection of more detailed data and/or a survey of the staff within each Trust.

Using the 2008 NHS reference costs[16] as a guide the one day decrease in LOS is equivalent to a saving of approximately £600 per patient; a considerable amount given the number of women undergoing breast surgery each year. In reality however, the majority of the costs are incurred in the first half of a hospital stay when assessment, intervention and input from staff are at a maximum, whilst decreases in LOS tend to result from a reduction in the second half of the stay which are cheaper[6]. Nevertheless, the trend towards shorter hospital stays allows more patients to be treated.

The median LOS was 5 days for mastectomy patients, but this varied from 3 to 8 days according to Trust. The median LOS for BCS patients was lower at 3 days, but the maximum was 7 days and there were some Trusts where the median was 0 or 1 day. Such variation reflects differing policies on the factors mentioned above. We are aware of some Trusts treating the majority of women undergoing BCS as day-cases and this will account for the low LOS in these Trusts. We are also aware that several Trusts have recently piloted 23-hour mastectomies, with patients being discharged without drains and followed up at home by nurses[17]. It has been shown that the use of drains does not prevent seroma formation and is associated with higher pain scores after surgery[18]. This change occurred after the end of our study period and would not have influenced our results, but we would expect LOS after mastectomy to continue to reduce as 23-hour mastectomies become more widespread.

There was also large variation in the proportion of women undergoing mastectomy by Trust, ranging from 14% to 80%. This variation is well documented in reports from the NHS Breast Screening Programme[19] and may be due to the extent to which surgical choice is offered and patients are made aware that it is their choice[20]. In this study, which includes symptomatic patients who generally have larger and more advanced cancer, this effect is probably magnified.

We used multilevel modelling to take account of the hierarchical nature of the data and to estimate the amount of unexplained variation attributable to each level. The Trust and surgical team level variances were small but statistically significant. We included consultant workload in the model, as a proxy for experience, but there was no significant relationship between workload and LOS. In addition, we included a measure of bed availability but this showed no association with LOS. However, these measures may have been too crude to show any association. The amount of variation was slightly higher at the Trust level than the surgical team level. It has been suggested that doctors working within the same hospital conform to the practice of immediate colleagues, resulting in smaller within doctor variation and larger within hospital variation[12]. It would appear that Trust policies, such as the uptake of early discharge, have a substantial effect on LOS, but such factors are not captured within routine data and, as mentioned earlier, would involve the collection of more detailed data tailored towards the answering of specific research questions. Such measures could be developed in future work, for example, guidelines for LOS and the categories of patients which can safely be treated as day cases, as well as the education of staff within breast teams, should help reduce the current level of variation.

In this study we excluded cases treated by surgeons with a workload less than 10 per year and mastectomy cases where the LOS was zero or one day, as it was felt that the data were unreliable and likely to have been recorded incorrectly. To test the effect of these assumptions the analysis was repeated including all cases but there was no substantial change to the fixed effects. The variances, however, were larger, particularly at the surgical team level, reflecting the amount of unexplained variation being added.

The factors affecting LOS are complex and not necessarily associated with quality of care, and there is little evidence of any relation between LOS and outcome[21]. In order to look further into this issue we also looked at the total number of days spent in hospital in the 30 days following the breast cancer surgery and attempted to identify admissions specifically related to the breast cancer. The median LOS in the 30 days was 4 days, very similar to the LOS for the initial surgery. According to the data 13% of women in this study were re-admitted for reasons related to their breast cancer, but the majority of these admissions were for women to receive chemotherapy, suggesting that few women are readmitted with complications related to their breast cancer surgery. However, it is unclear how complete and accurate the coding of secondary diagnoses in HES is and so it is possible that these figures are an underestimate.

## Conclusion

In summary, the number of days spent in hospital after surgery for breast cancer has continued to decline for several decades. It is likely that the reductions in LOS are partly due to changes in surgical technique (increasing proportions of BCS and SLNB) and partly due to changes in practice (admission on day of surgery and earlier discharge). However, this study has identified wide variation in practice with substantial cost implications for the NHS. Further work is required to explain the present situation of substantial variation of LOS amongst Trusts and surgical teams.

## Abbreviations

BCS: Breast conserving surgery; FCE: Finished consultant episode; HES: Hospital episode statistics; LOS: Length of stay; LOSR: Length of stay ratio; MREC: Multi-centre Research Ethics Committee; NIGB: National Information Governance Board; SLNB: Sentinel lymph node biopsy

## Competing interests

The authors declare that they have no competing interests.

## Authors' contributions

AD carried out the analysis and wrote the initial draft of the manuscript. JD linked and managed the data. RMW provided statistical support. ML and GL helped with interpretation of the data and results. DF conceived the study and helped draft the manuscript. All authors contributed to the final draft of the manuscript. All authors read and approved the final manuscript.

## Pre-publication history

The pre-publication history for this paper can be accessed here:



## References

[B1] UK Parliament (1983). Written Answers: NHS Hospitals (Inpatients). http://hansard.millbanksystems.com/written_answers/1983/nov/16/nhs-hospitals-inpatients.

[B2] Organisation for Economic Co-operation and Development (2007). Health at a glance 2007: OECD indicators.

[B3] Shi L (1996). Patient and hospital characteristics associated with average length of stay. Health Care Management Review.

[B4] Burns LR, Wholey DR (1991). The effects of patient, hospital and physician characteristics on length of stay and mortality. Medical Care.

[B5] Morgan M, Beech R (1990). Variations in lengths of stay and rates of day case surgery: implications for the efficiency of surgical management. Journal of Epidemiology and Community Health.

[B6] Clarke A (1996). Why are we trying to reduce length of stay? Evaluation of the costs and benefits of reducing time in hospital must start from the objectives that govern the change. Quality in Health Care.

[B7] Roos NP, Flowerdew G, Wajda A (1986). Variation in physicians' hospitalisation practices: A population-based study in Manitoba, Canada. American Journalof Public Health.

[B8] Neutel CI, Gao RN, Gaudette L, Johansen H (2004). Shorter hospital stays for breast cancer. Health Reports.

[B9] Wilk EA van der (2009). Trend in ALOS for Breast cancer (ICD-10 code: C50), in selected countries, 1996-2005. http://www.euphix.org/object_document/o5387n27121.html.

[B10] Lindqvist R, Moller TR, Stenbeck M, Diderichsen F (2002). Do changes in surgical procedures for breast cancer have consequences for hospital mean length of stay?. International Journal of Technology Assessment in Health Care.

[B11] Bundred N, Maguire P, Reynolds J, Grimshaw J, Morris J, Thomson L (1998). Randomised controlled trials of effects of early discharge after surgery for breast cancer. British Medical Journal.

[B12] Westert GP, Nieboer AP, Groenewegen PP (1993). Variation in duration of hospital stay between hospitals and between doctors within hospitals. Social Science & Medicine.

[B13] Charlson ME, Pompei P, Ales KL, McKenzie CR (1987). A new method of classifying prognostic comorbidity in longitudinal studies: development and validation. J Chron Dis.

[B14] Department of Health (2009). Bed availability and occupancy, NHS Organisations in England, 2005-06. Hospital Activity Statistics.

[B15] Rasbash J, Browne WJ, Healy M, Cameron B, Charlton C (2005). MLwiN version 2.02.

[B16] Department of Health (2008). NHS trust reference cost schedules 2006/07.

[B17] NHS Improvement Programme (2008). Cancer Improvement. Winning Principle 2: Case Studies.

[B18] Jain PK, Sowdi R, Anderson ADG, MacFie J (2004). Randomized clinical trial investigating the use of drains and fibrin sealant following surgery for breast cancer. British Journal of Surgery.

[B19] NHS Breast Screening Programme & Association of Breast Surgery at BASO (2009). An audit of screen-detected breast cancers for the year of screening April 2007 to March 2008. Sheffield, NHS Cancer Screening Programmes.

[B20] Ballinger RS, Fortes Mayer K, Lawrence G, Fallowfield L (2008). Patients' decision-making in a UK specialist centre with high mastectomy rates. The Breast.

[B21] Clarke A (2002). Length of in-hospital stay and its relationship to quality of care. Quality and Safety in Health Care.

